# Tanshinone IIA Improves Acute Gouty Arthritis in Rats through Regulating Neutrophil Activation and the NLRP3 Inflammasome

**DOI:** 10.1155/2022/5851412

**Published:** 2022-12-19

**Authors:** Lianjie Xu, Xiao Liu, Yurong Zhang, Tao Jia, Limei Li, Yan Du, Wenhui Chen, Shan Zhang

**Affiliations:** ^1^Faculty of Basic Medicine, Yunnan University of Traditional Chinese Medicine, Kunming, Yunnan 650500, China; ^2^Qujing Hospital of Traditional Chinese Medicine, Qujing, Yunnan 655000, China; ^3^Department of Orthopedics, First Clinical Medical College of Yunnan University of Traditional Chinese Medicine, Kunming, Yunnan 650021, China; ^4^Yunnan Provincial Key Laboratory of Molecular Biology for Sinomedicine, Kunming, Yunnan 650500, China

## Abstract

**Objectives:**

To investigate the prevention and treatment effect of tanshinone IIA (TIIA) on acute gouty arthritis (AGA) and its mechanism.

**Methods:**

The anti-AGA effect of TIIA was observed *in vivo* and *in vitro*. Neutrophils were isolated from the abdominal cavity of mice, and the anti-AGA effect of TIIA was investigated in a rat model of MSU-induced AGA. The pathological changes of the ankle joint tissues were assessed by H&E. Cytokine and chemokine expression were determined by ELISA and RT-qPCR. The NLRP3 inflammasome pathway protein levels in the ankle joint tissues were evaluated via western blotting. Neutrophil migration was evaluated in air pouch and transwell assays. Immunohistochemistry and immunofluorescence analysis evaluate the release of myeloperoxidase (MPO), neutrophil elastase (NE), and citrullination of histone H3 (CitH3). Beclin-1 and LC3B expressions were determined using western blotting and immunofluorescence. *Key Findings*. Treatment with TIIA alleviated synovial hyperplasia and neutrophil infiltration, regulated cytokine and chemokine expressions, and inhibited NLRP3 activation in AGA rats, neutrophil migration, MPO, NE, and CitH3 expression, and LC3B and Beclin-1 protein expression.

**Conclusions:**

These results demonstrate that TIIA can effectively enhance AGA by focusing on the neutrophils and NLRP3 inflammasome, demonstrating that TIIA may act as a potential helpful agent for AGA.

## 1. Introduction

Gout is a common arthritis disease that is mainly characterized by monosodium urate (MSU) deposition in the synovium or periarticular tissues [1]. Acute gouty arthritis (AGA) is the most common symptom of gout. The main clinical manifestations include sudden local redness, swelling, and pain. If not actively prevented or treated, it can lead to joint deformity and dysfunction. In spite of the fact that the etiology and pathogenesis of this malady have not been completely clarified, joint irritation and damage to AGA are known to be intervened by the influx of innate immune cells into the joint space [2].

Inflammasome assembly and activation are important processes in innate immune defense. Recent studies have shown a positive correlation between AGA progression and NOD-like receptor family 3 (NLRP3) inflammasome activation [3]. Hao et al. [4] showed that MSU crystals can increase NLRP3 inflammasome activation, resulting in ankle swelling and severe synovial inflammation in AGA rats, accompanied by obvious neutrophil infiltration. MSU binds to the NLRP3 inflammasome, resulting in cysteine protease-1 (caspase-1) activation, promoting interleukin-18 (IL-18) and IL-1*β* production, and promoting neutrophil recruitment [5]. Neutrophils are the first line of defense of the innate immune system and are considered one of the major factors in the acute inflammatory response [6]. IL-1*β* production is considered an initiating cause that promotes gouty inflammation and the recruitment of large numbers of neutrophils at inflammation sites, which lead to amplification of the joint inflammatory response [5]. Neutrophils are rarely observed in the synovial fluid of healthy subjects, whereas patients of AGA show excessive accumulation and infiltration in the synovial fluid and joint cavity [7]. Neutrophils at the site of joint inflammation ingest small MSU crystals and include them in the phagosomes. The acidic environment and reactive oxygen species in phagosomes affect MSU [8]. Activated neutrophils can produce and release IL-6, tumor necrosis factor-*α* (TNF-*α*), IL-8, and other proinflammatory cytokines [9], neutrophil elastase (NE), and myeloperoxidase (MPO) [10]. These enzymes degrade the extracellular matrix in the joint structure and recruit other immune cells to damage the tissue or interact with other cell populations. MSU can also trigger neutrophils to form neutrophil extracellular traps (NETs), structures composed of depolymerized DNA extracellular chains, and neutrophil granule proteins that are also involved in the occurrence and development of AGA [11]. Therefore, neutrophils are important targets for AGA treatment.

Tanshinone IIA (TIIA) is one of the main active components in *Salvia miltiorrhiza* (Dan Shen). It can also exert anti-inflammatory effects in many tissues. TIIA can significantly decrease IL-1*β*, TNF-*α*, and inducible nitric oxide synthase in a rat model of osteoarthritis [12]. In lipopolysaccharide-induced acute lung injury, TIIA can significantly reduce the infiltration of neutrophils, MPO activity, and inflammatory cytokine expression in bronchoalveolar lavage fluid [13]. In addition, TIIA inhibits neutrophil migration and aggregation in inflammatory sites, inhibits their activation, promotes the timely apoptosis of activated neutrophils, inhibits NET formation, and reduces the inflammatory response and tissue damage in rheumatoid arthritis [14]. However, whether TIIA can ameliorate inflammation in AGA remains unclear.

Herein, we aimed to elucidate the molecular mechanism of action of TIIA in the treatment of AGA in rat and cell models. Our study manifested that TIIA significantly reduced ankle circumference in AGA rats. TIIA inhibited protein expression in the NLRP3 inflammasome pathway, thereby inhibiting neutrophil infiltration in the joints and periarticular tissues. TIIA inhibited neutrophil migration *in vivo* and *in vitro*. Furthermore, TIIA suppressed NET formation via inhibiting autophagy. Therefore, our findings offer insights into the therapeutic potential of TIIA on AGA that acts by targeting neutrophil activity and NLRP3 inflammasome pathway.

## 2. Materials and Methods

### 2.1. Animals

Eight-week-old male SD rats were purchased from the Experimental Animal Center of Kunming Medical University (Kunming, Yunnan, China) (SCXK (Dian) 2020-0004). Rats were fed under standard environmental conditions (23 ± 2°C, 60% humidity, and 12/12 h light/dark cycles). All animals received humane care according to the institutional animal care guidelines approved by the Experimental Animal Ethics Committee of Kunming Medical University (No. KMMU2021722).

### 2.2. Reagents

TIIA sulfonate was purchased from the First Biochemical Pharmaceutical Corporation of Shanghai (cat. no. H31022558). MSU (cat. no. U2875-5 g) and TIIA (cat. no. T4952-5 mg) were purchased from Sigma. UA (cat. no. C012-2-1) test kits were purchased from Nanjing Jiancheng Bioengineering Institute. XOD (cat. no. MM-0725R1), TNF-*α* (cat. no. MM-0180R1), IL-6 (cat. no. MM-0190R1), IL-8 (cat. no. MM-0175R1), CXCL1 (cat. no. MM-70081R1), CXCL2 (cat. no. MM-70272R1), S100A8A9 (cat. no. MM-0999R1), IL-1RA (cat. no. MM-0225R1), IL-1*β* (cat. no. MM-0047R1), and IL-18 (cat. no. MM-0194R1) ELISA assay kits were purchased from Meimian Biotechnology (Jiangsu, China). Primary anti-bodies: MPO (cat. no. ab9535), NE (cat. no. ab21595), CitH3 (cat. no. ab5103), and LC3B (cat. no. ab48394) were purchased from Abcam, Inc. (MA, USA); Beclin-1 (cat. no. 3495S) was purchased from CST; antibodies against NLRP3 (cat. no. DF7438), apoptosis-related speckle-like protein (ASC) (cat. no. DF6304), and caspase-1 (cat. no. 22915-1-AP), IL-1*β* (cat. no. 26048-1-AP) were, respectively, purchased from Affinity (Affinity Biosciences, USA) and Proteintech. TRIzol® was purchased from Invitrogen, Thermo Fisher Scientific, Inc. (cat. no. 15596026). ReverTra Ace®qPCR RT Master Mix with gDNA Remover and SYBR-Green Master Mix kit were purchased from Toyobo Life Science (cat. no. QPK201), and the primers for the target genes were synthesised from Sangon Biotech Co., Ltd. (Shanghai, China).

### 2.3. Rat Model of MSU-Induced AGA

In this experiment, the AGA rat model was replicated via intra-articular injection of MSU, as designed by Coderre et al. (1987). Rats were acclimatized for seven days before the start of the experiment and were randomly divided into four groups (*n* = 10 per group): control, TIIA, AGA, and AGA+TIIA groups. Sodium TIIA sulfonate (20 mg/kg/day) was injected intraperitoneally in the TIIA and AGA+TIIA groups for seven days, and the same volume of normal saline was injected intraperitoneally in the control and AGA groups. One hour after administration on the 5^th^ day, 0.2 mL and 25 mg/mL MSU was injected into the right ankle cavity of rats in the AGA and AGA+TIIA groups for model preparation. The control and TIIA groups were injected with the same volume of normal saline.

### 2.4. Histological Analysis

The rat ankle joint tissues were fixed in 4% paraformaldehyde for 48 h, decalcified in EDTA solution for approximately three months, embedded in paraffin wax, and cut into 4 *μ*m thick slices, and then, ankle joint slices were stained with haematoxylin-eosin (H&E). The images were captured using SQS-1000SQS-1000 image system.

### 2.5. Immunohistochemistry

Ankle joint tissue (5 *μ*m thick) of paraffin areas was cut for immunohistochemical analysis. The tissue sections were dewaxed with xylene and dehydrated with ethanol, after inundation in citric acid antigen solution at a pH of 6.0, after PBS washes, and blocked endogenous peroxidase activity with 3% H_2_O_2_ for 15 min. Next, the sections were incubated with anti-MPO (1 : 40) and anti-NE (1 : 500) antibodies at 4°C for overnight. The Goat Anti-Rabbit IgG secondary antibodies were applied to the first antibody incubation and then stained with DAB, the substrate of chromogenic peroxidase. The sections were stained with haematoxylin, and the images were captured using SQS-1000SQS-1000 image system.

### 2.6. Mouse Air Pouch Experiments

Pathogen-free 8-week-old male C57BL/6 mice (6/group) were randomly assigned to four groups: control, TIIA, AGA, and AGA+TIIA. After the mice were anesthetized with sodium pentobarbital injection, 3 mL sterilized air was injected subcutaneously into the back to form an air bag on days 0 and 3. On the 6^th^ day, 1 mL HBSS and 200 *μ*L 25 mg/kg of MSU with or without 30 mg/kg TIIA were injected into the air pouches of mice 6 h before the mice were sacrificed by inhalation of ether asphyxiation. The mice were lavaged twice with 3 mL HBSS buffer to collect the cells in the air bag. The collected lavage solution was centrifuged for 10 min. After discarding the supernatant, the cells were resuspended for cell counting.

### 2.7. Neutrophil Preparation and Culture

One milliliter of 10% protease peptone was injected intraperitoneally 12 and 24 h before the mice were sacrificed via sodium pentobarbital. The abdominal cavities of mice were lavaged with 5 mL of RPMI-1640 comprising 10% FBS. After 5 min, the peritoneal lavage solution was extracted with a syringe into a 15 mL centrifuge tube, filtered with a cell filter screen, and centrifuged for 5 min. The supernatant was removed, and the cells were resuspended in the prepared neutrophil complete medium. Neutrophils were treated by MSU (500 *μ*g/mL) and TIIA (20 *μ*mol/L or 40 *μ*mol/L).

### 2.8. Real-Time Quantitative PCR (RT-qPCR) Analysis

2 mL neutrophils were incubated in 6-well plates at a density of 5 × 10^6^ cells/mL per well. Cells were cultured and treated with different concentrations of TIIA and MSU (500 *μ*g/mL) at 37°C for 2 h. Total RNA was extracted using TRIzol (Invitrogen) and reverse transcribed into cDNA using the ReverTra Ace qPCR RT kit (ToYoBo). RT-qPCR was performed in a Roche LightCycler 96 analyser using 96-well reaction plates, pairs of oligonucleotide primers, and SYBR green Premix EX Taq™ II for TNF-*α* (sense: ACAGAAAGCATGATCCGCG, antisense: GCCCCCCATCTTTTGGG); IL-8 (sense: CAAGGCTGGTCCATGCTCC, antisense: TGCTATCACTTCCTTTCTGTTGC); IL-1*β* (sense: GCAACTGTTCCTGAACTCAACT, antisense: ATCTTTTGGGGTCCGTCAACT); IL-1RA (sense: GCTCATTGCTGGGTACTTACAA, antisense: CCAGACTTGGCACAAGACAGG); CXCL1 (sense: CTGGGATTCACCTCAAGAACATC, antisense: CAGGGTCAAGGCAAGCCTC); CXCL2 (sense: CCAACCACCAGGCTACAGG, antisense: GCGTCACACTCAAGCTCTG); *β*-actin (sense: TATCGGACGCCTGGTTA, antisense: TGTGCCGTTGAACTTGC). The relative expression level of mRNA was calculated using 2^−ΔΔCt^. The primers for the target genes were synthesised by Sangon Biotech Co., Ltd. (Shanghai, China).

### 2.9. ELISA Analysis

UA, XOD, TNF-*α*, IL-6, IL-8, CXCL1, CXCL2, S100 calcium-binding protein A8A9 complex (S100A8A9), IL-1RA, IL-1*β*, and IL-18 in serum were measured by ELISA kits (Mei mian, Jiangsu, China) based on the manufacturer's instructions. The OD value was measured at 450 nm using a Spark 10 M Multimode reader (Tecan); then, calculate the concentrations of the aforementioned indicators.

### 2.10. Immunofluorescence Assay

Neutrophils were seeded at a density of 10^6^ cells/plate. The cells were fixed for 20 min in 4% paraformaldehyde and permeabilized by adding 0.5% Triton X-100 for 20 min. Then, drop an appropriate amount of bovine serum albumin (5% BSA) into the dish to block, remove the serum after blocking, and directly incubate with the diluted anti-NE and anti-MPO at 4°C overnight. After washing, secondary antibodies with fluorescein were added and incubated for 1 h and then counterstained with 4′,6-diamino-2-phenylindole (DAPI) at room temperature for 5 min. Cells were imaged using a confocal microscope (BX60, Olympus, Tokyo, Japan).

### 2.11. Western Blot

Samples were lysed in RIPA lysis buffer and PMSF (Beyotime, China). Total protein lysates were separated using gradient gel electrophoresis (10%, PAGE Gel Fast Preparation Kit, Epizyme), and the separated proteins were subsequently transferred to a PVDF membranes, blocked with 5% skimmed milk for 2 h at room temperature, and blotted overnight at 4°C with primary antibodies against NLRP3, ASC, caspase-1, IL-1*β*, Beclin-1, and LC3B. The membranes were incubated with a goat anti-rabbit horseradish peroxidase-conjugated IgG secondary antibody at room temperature for 4 h. The ECT reagent was used to obtain the protein bands. The intensities of protein bands were quantified using the ImageJ software.

### 2.12. Transwell Analysis

The collected neutrophils were diluted to 5 × 10^6^/mL with neutrophil complete medium, and 200 *μ*L of the cell suspension was inoculated into the upper chamber of the transwell with or without MSU (500 *μ*g/mL) and TIIA (20 *μ*mol/L or 40 *μ*mol/L). Then, add 600 *μ*L of neutrophil complete medium in the lower chamber and incubated in the incubator for 30 min; and the liquid was collected in the lower chamber, and the neutrophils were counted.

### 2.13. Statistical Analysis

Result analysis and mapping were performed using SPSS 25.0, GraphPad Prism 8.0, and the experimental data were expressed as mean ± standard deviation (SD). One-way analysis of variance was used to compare multiple groups, and Dunnett's test was used to compare two samples. Differences were considered statistically significant at *P* < 0.05.

## 3. Results

### 3.1. TIIA Alleviated AGA in Rats

**Figure 1:**
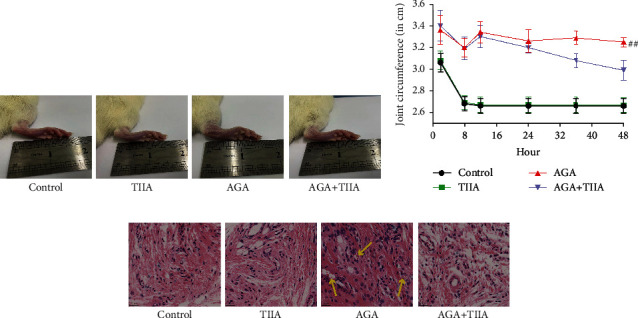
TIIA treatment alleviates MSU-induced AGA in rats. (a) The degree of swelling of right ankle joints was observed at 48 h after MSU induction. (b) The circumference of the ankle joints of rats. ^##^*P* < 0.01 when comparing the AGA group with the control (*n* = 8). (c) H&E staining of the ankle joint sections, the arrow indicates pannus formation, original magnification: ×400.

We used an MSU-induced AGA rat model to explore the therapeutic effects of TIIA. We found that the MSU group had higher degree ankle joint swelling and joint circumference when compared to the control group, but the degree of joint swelling (Figure 1(a)) and joint circumference (Figure 1(b)) of the AGA+TIIA group significantly were reduced. H&E staining revealed synovial hyperplasia, massive inflammatory cell infiltration, and pannus formation in the ankle joints sections of MSU-induced AGA compared with control rats. Treatment with TIIA significantly alleviated arthritis in AGA rats (Figure 1(c)).

### 3.2. Effect of TIIA on UA, Cytokines, and Chemokines In Vivo and In Vitro

**Figure 2:**
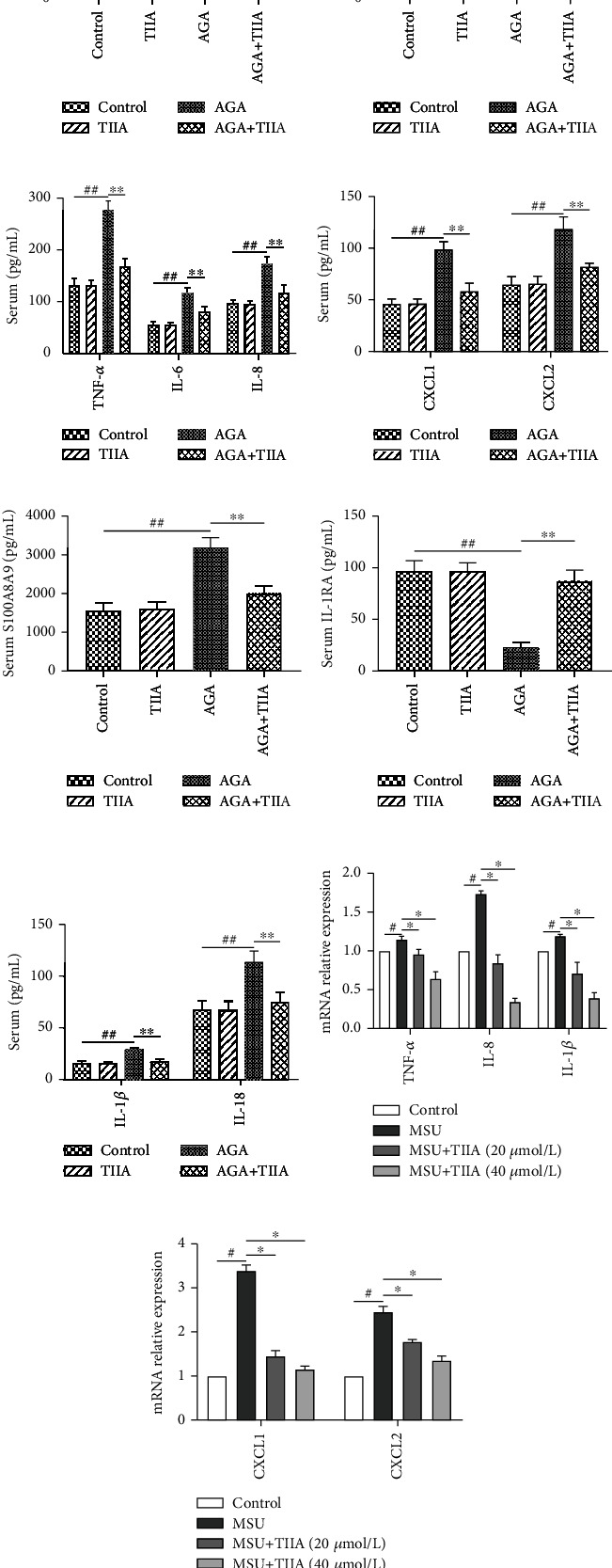
TIIA decreases UA, cytokine, or chemokine production in serum and neutrophils *in vitro*. (a–g) ELISA analyses of serum UA, XOD, IL-6, TNF-*α*, IL-8, CXCL1, CXCL2, S100A8A9, IL-1*β*, IL-18, and IL-1RA expressions in the MSU-induced AGA rats (*n* = 6). (h, i) QRT-PCR analyses of IL-1*β*, IL-6, TNF-*α*, CXCL1, and CXCL2 mRNA levels in neutrophils *in vitro* (*n* =6). ^#^*P* < 0.05 and ^##^*P* < 0.01, when compared to the control; ^∗^*P* < 0.05 and ^∗∗^*P* < 0.01, when compared to the AGA.

Next, we investigated whether TIIA could regulate UA, cytokine, and chemokine expressions in MSU-induced AGA. ELISA results showed that MSU-induced rats had significantly increased serum UA, XOD, IL-6, TNF-*α*, IL-8, CXCL1, CXCL2, S100A8A9, IL-1*β*, and IL-18, as well as reduced IL-1RA, compared with control rats, but TIIA treatment reversed their expression in the serum of AGA rats (Figures 2(a)–2(g)). In addition, TIIA significantly downregulated IL-8, IL-1*β*, TNF-*α*, CXCL1, and CXCL2 mRNA levels in a dose-dependent manner by qRT-PCR analysis *in vitro* (Figures 2(h) and 2(i)).

### 3.3. TIIA Inhibited the NLRP3 Inflammasome Pathway

**Figure 3:**
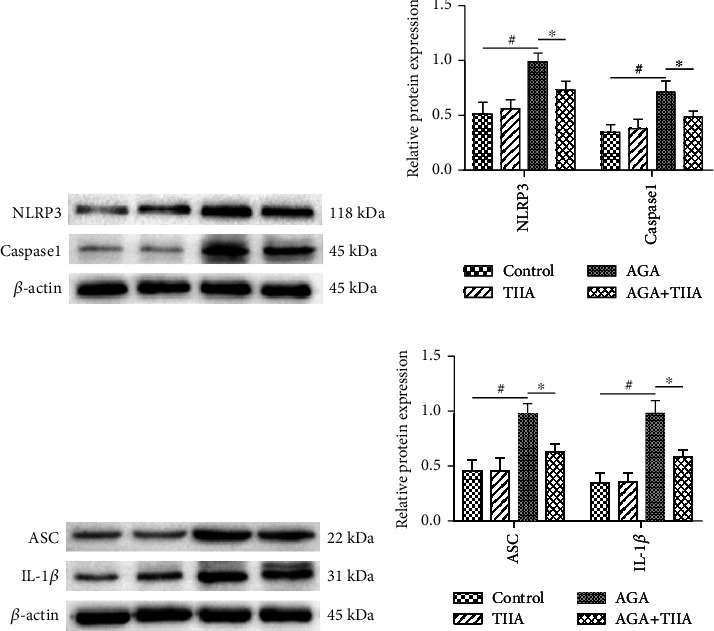
TIIA inhibited protein expression in the NLRP3 inflammasome pathway. (a) Western blotting analysis of NLRP3 and caspase-1 in ankle joints. (b) Density analyses of NLRP3 and caspase-1 expression in (a). (c) Western blotting analysis of ASC and IL-1*β* in ankle joints. (d) Density analyses of ASC and IL-1*β* expression in (c) (*n* = 3). AGA vs. control, ^#^*P* < 0.05; TIIA vs. AGA, ^∗^*P* < 0.05.

With an in-depth understanding of the pathological mechanism of AGA, MSU-induced NLRP3 inflammasome activation was found to play pivotal role in progression of AGA [15]. To further investigate the effect of TIIA on the NLRP3 inflammasome pathway, we examined the expression of NLRP3, caspase-1 (Figures 3(a) and 3(b)), ASC, and IL-1*β* (Figures 3(c) and 3(d)) in the ankle joints of rats using western blotting. Compared with the control group, their expression in the AGA group was significantly increased, while TIIA expression was reduced.

### 3.4. TIIA Inhibited MSU-Induced Neutrophil Migration

**Figure 4:**
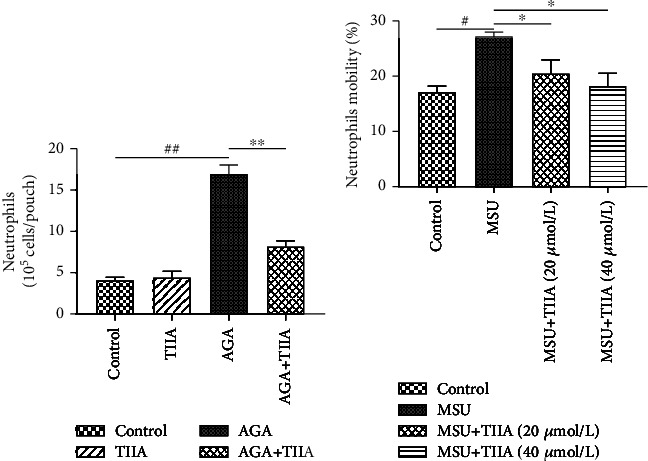
TIIA inhibited MSU-induced neutrophil migration. We administer MSU or TIIA into the air pouches. Exudates were collected after 6 h, and neutrophils were numbered and identified via cytology as described in Section 2.6. (a) The number of neutrophils in mouse air pouches (*n* = 6), AGA vs. control, ^##^*P* < 0.01; AGA+TIIA vs. AGA, ^∗∗^*P* < 0.01. (b) Transwell analysis results of *in vitro* neutrophil mobility (*n* = 6), AGA vs. control, ^#^*P* < 0.05; MSU*+*TIIA vs. AGA, ^∗^*P* < 0.05.

The effect of TIIA on neutrophil migration was investigated by air pouch experiments in mice (Figure 4(a)). Treatment with TIIA notably reduced the number of neutrophils. In addition, we examined the regulatory impact of TIIA on neutrophil migration *in vitro* via a transwell assay (Figure 4(b)). MSU-induced cells promoted the transmembrane migration of neutrophils when compared to the control group, whereas TIIA reversed this trend and inhibited their migration.

### 3.5. TIIA Inhibits MPO, NE, and CitH3 Expression In Vivo and In Vitro

**Figure 5:**
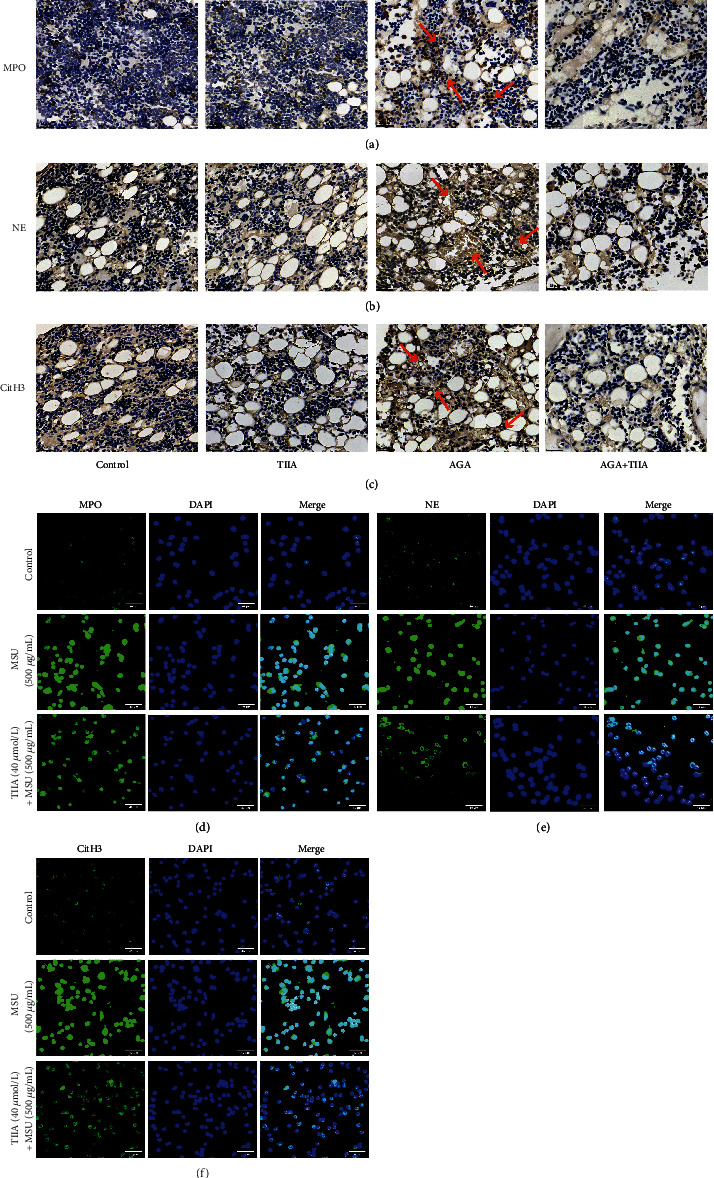
TIIA inhibited the formation of MPO, NE, and CitH3. (a–c) Release of MPO, NE, and CitH3 by immunohistochemistry analysis *in vivo*, original magnification: ×400. Arrows indicate positive signals. (d–f) Immunofluorescence analyses of MSU-induced MPO, NE, and CitH3 expression *in vitro*.

NET formation and release are considered defense mechanisms against pathogens or endogenous danger signals and are related to the inflammatory response [16]. Excessive NET formation leads to deterioration of inflammation and the occurrence of autoimmune diseases [17, 18]. To investigate whether TIIA inhibited NET formation, we tested the levels of MPO, NE, and CitH3 in the ankle joints of AGA rats by immunohistochemical analysis. The levels of MPO, NE, and CitH3 in the AGA group increased significantly, when compared to the control. However, TIIA significantly inhibited MPO, NE, and CitH3 expression (Figures 5(a)–5(c)). In addition, we obtained that TIIA can inhibit the expression of MPO, NE, and CitH3 by immunofluorescence assay *in vitro* (Figures 5(d)–5(f)).

### 3.6. TIIA Inhibited Autophagy of MSU-Induced Neutrophils

**Figure 6:**
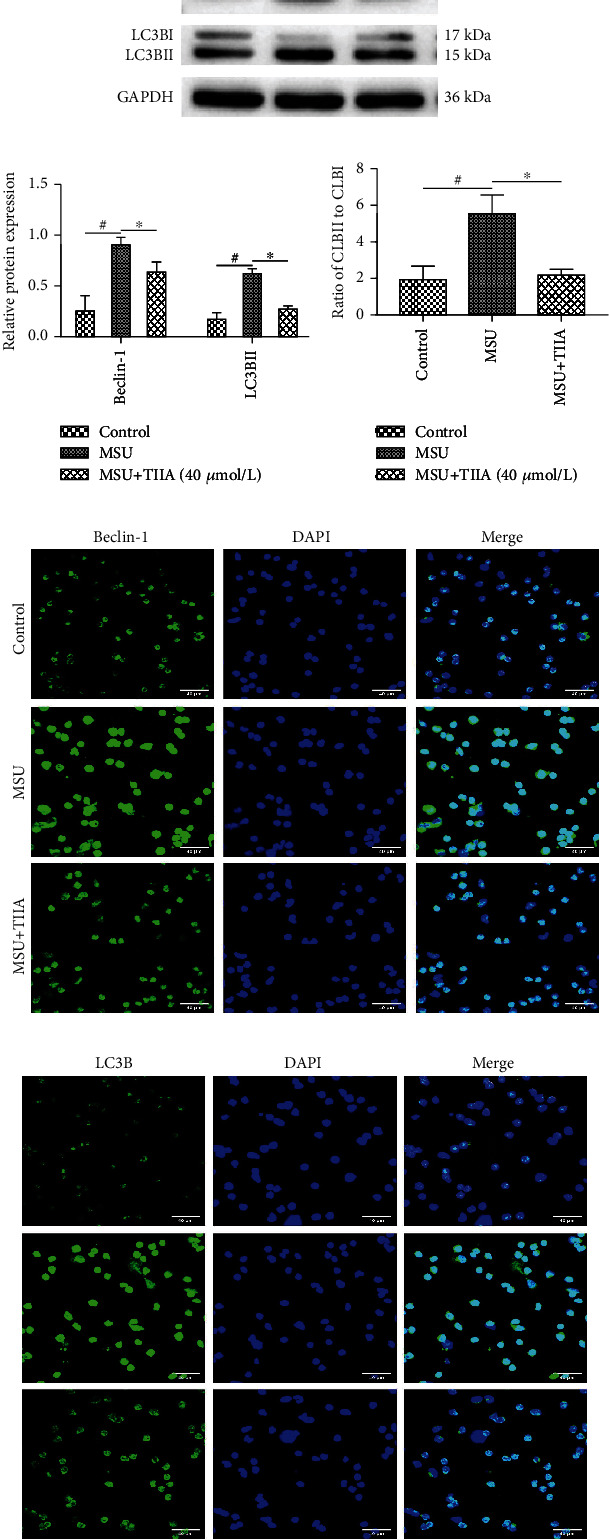
TIIA inhibits autophagy in MSU-induced neutrophils. (a) The neutrophils of different groups were analysed for Beclin-1 and LC3B protein expression via western blotting (*n* = 3). (b) Quantification of Beclin-1 and LC3BII shown in (a). (c) Ratio of LC3BII over LC3BI expression. AGA vs. control, ^#^*P* < 0.05; TIIA vs. AGA, ^∗^*P* < 0.05. (d, e) Immunofluorescence analyses of MSU-induced LC3B and Beclin-1 levels *in vitro*.

LC3B-I to LC3B-II conversion is an important marker of autophagy activation [19]. In order to investigate whether TIIA can resolve inflammation by inhibiting neutrophil autophagy, the neutrophils of different groups were analysed for Beclin-1 and LC3B protein expression via western blotting (Figures 6(a) and 6(b)). TIIA treatment significantly reduced Beclin-1, LC3BII expression, and the ratio of LC3BII to LC3BI (Figure 6(c)). In addition, neutrophil autophagy was also examined via immunofluorescence analysis (Figures 6(d) and 6(e)). Compared with the control group, MSU-induced cells promoted neutrophil autophagy, which was inhibited by TIIA.

## 4. Discussion

AGA presents as intermittent episodes of severe painful arthritis caused by the innate immune response to MSU crystals [20]. After immune cells including macrophages and dendritic cells are activated, neutrophils influx into the synovium of the affected joint [21]. At the onset of AGA joint inflammation, more than 80% of neutrophils infiltrate the synovium and synovial fluid, whereas they are not present in normal joints synovial fluid. Phagocytosis of MSU crystals by neutrophils in the synovial fluid is a marker for AGA diagnosis [22]. Once neutrophils enter the joints, they release a series of inflammatory mediators, leading to joint inflammation and pain [23]. Our study confirmed that TIIA reduced neutrophil infiltration in inflammatory tissues in a rat model of AGA and in an *in vitro* transwell experiment. We also found that TIIA could inhibit neutrophil migration in a model of balloon inflammation.

Neutrophils infiltrating the inflammatory site in the joint are activated after recognizing MSU, and they release various mediators, such as proinflammatory factors (TNF-*α*, IL-6, and IL-18), anti-inflammatory factors (IL-1RA), and chemokines (CXCL1 and CXCL2), which cause tissue damage in aseptic inflammation [24]. S100A8 and S100A9 are small cell solute proteins in the form of homodimers; however, the presence of Ca increases the formation of the heterologous complex S100A8/A9, which can promote neutrophil migration and recruitment [25]. IL-8 (homologous ligand of CXCR2) is an inflammatory chemokine with special affinity for neutrophils activation and recruitment at the site of injury [26]. A previous study found that TNF-*α*, IL-1*β*, and IL-8 are involved in AGA initiation and act as inducers of persistent inflammation [27]. Thus, inhibition of inflammatory mediators can reduce neutrophil recruitment and pain. Our study showed that TIIA significantly enhanced IL-1RA expression and reduced IL-6, TNF-*α*, IL-8, CXCL1, CXCL2, S100A8A9, IL-1*β*, and IL-18 expression and secretion.

Inflammasome bodies are macromolecular multiprotein complexes that regulate proinflammatory cytokines at the molecular level when cells are infected or start a stress response. They are composed of NLRP3, ASC, and pro-caspase-1 [28]. In the early stages of AGA attack, monocytes enter the inflammatory site and gradually develop into mature macrophages to phagocytize MSU crystals. Activation of the NLRP3 inflammasome triggers NLRP3-ASC-pro-caspase-1 assembly, leading to activation of pro-caspase-1 to produce active caspase-1. Active caspase-1 then catalytically shears pro-IL-1*β* and pro-IL-18, enabling it to produce IL-1*β* and IL-18 with mature and biological activity [29]. Mature IL-1*β* binds to the IL-1*β* receptor in synovial cells to activate NF-*κ*B. This starts and regulates the gene expression of many inflammatory mediators, and adhesion molecules related to inflammatory immune responses, including IL-1*β*, IL-8, TNF-*α*, and neutrophil chemokines, resulting in an inflammatory cascade. Many neutrophils infiltrate the joint MSU deposition site, which can enhance IL-1*β* binding to its receptor, promoting the invasion of more neutrophils and aggravating the inflammatory reaction in the joint [30, 31]. Jorgensen et al. [32] found that IL-18 can affect NET formation by regulating neutrophils. In addition, IL-1*β* and IL-18 can attract neutrophils and induce them to release NETs in the damaged tissues. In turn, enhanced NET formation can lead to an increase in NLRP3 inflammatory body activation and trigger IL-18 and IL-1*β* synthesis, leading to the inflammatory cycle [33]. The findings of the present study demonstrated that TIIA significantly inhibited NLRP3 inflammasome and NET formation.

Autophagy degrades cytoplasmic proteins and organelles and plays a very important role in stabilizing the intracellular environment [34, 35]. In neutrophils, autophagy can promote NET formation [36]. Inhibition of autophagy attenuates LC3 expression and significantly reduces NET formation [37]. When inflammation occurs in the body, neutrophils are recruited to sites of inflammation to induce autophagy. They phagocytize and kill microorganisms through the formation of autophagic lysosomes and fusion of phagosomes and particles [38]. In AGA, after MSU induces neutrophil autophagy, the ingested MSU promotes the release of lysosomal content by destroying the lysosomal membrane. The excessive release of neutrophil lysosomal content and the abnormal production and secretion of cytokines and inflammatory chemokines are the main causes of tissue damage [39]. Therefore, inhibiting neutrophil autophagy is essential to resolving inflammation. Our data revealed that TIIA treatment inhibited neutrophil autophagy by reducing Beclin-1 and LC3B activities.

Excessive purine intake and UA synthesis can further promote hyperuricemia. When the blood UA level of patients with hyperuricemia increases, MSU crystals are deposited in the joint cavity or around the joint, which further induces severe pain and inflammatory reactions, leading to gout [40]. Thus, inhibiting UA formation is very important in preventing AGA joint injury. XOD is a multifunctional molybdenum protein that catalyzes hypoxanthine and xanthine to form UA [41]. Kim et al. [42] found that a *S. miltiorrhiza* extract can reduce UA level in serum and can further reduce the pain response to inflammation. Our data revealed that TIIA significantly reduced serum UA levels by inhibiting XOD activity.

## 5. Conclusions

Our data showed that TIIA ameliorates inflammation in MSU-induced AGA by targeting the NLRP3 inflammasome and neutrophil activity. TIIA regulates cytokine and chemokine expression, thereby inhibiting neutrophil activation. This phytomedicine inhibited MPO and NE release by suppressing the NLRP3 inflammasome and neutrophil autophagy, thereby reducing tissue damage and inflammation. These data suggest the promising applicability of TIIA as a therapeutic agent for AGA treatment.

## Data Availability

The data used to support the findings of this study are included within the article.
